# Soil methane emissions from plain poplar (*Populus* spp.) plantations with contrasting soil textures

**DOI:** 10.1038/s41598-024-65300-0

**Published:** 2024-06-24

**Authors:** Xuehong Ma, Huili Feng, Jiahuan Guo, Changhui Peng, Daniel Kneeshaw, Weifeng Wang

**Affiliations:** 1 Co-Innovation Center for Sustainable Forestry in Southern China, College of Ecology and Environment, Nanjing Forestry University, Nanjing, 210037 China; 2 Key Laboratory of Ministry of Education for Genetics and Germplasm Innovation of Tropical Special Trees and Ornamental Plants, School of Tropical Agriculture and Forestry (School of Agricultural and Rural Affairs, School of Rural Revitalization), Hainan University, Haikou, 570228 China; 3 College of Geographic Science, Hunan Normal University, Changsha, 410081 China; 4 Department of Biological Sciences, University of Quebec at Montreal, Montreal, H3C 3P8 Canada

**Keywords:** Poplar plantations, Soil CH_4_ flux, Soil textures, Diurnal patterns, Environmental factors, Climate sciences, Ecology, Environmental sciences, Climate-change ecology, Forest ecology

## Abstract

The forest soil methane (CH_4_) flux exhibits high spatiotemporal variability. Understanding these variations and their driving factors is crucial for accurately assessing the forest CH_4_ budget. In this study, we monitored the diurnal and seasonal variations in soil CH_4_ fluxes in two poplar (*Populus* spp.) plantations (Sihong and Dongtai) with different soil textures using the static chamber-based method. The results showed that the annual average soil CH_4_ flux in the Sihong and Dongtai poplar plantations was 4.27 ± 1.37 kg CH_4_-C ha^–1^ yr^–1^ and 1.92 ± 1.07 kg CH_4_-C ha^–1^ yr^–1^, respectively. Both plantations exhibited net CH_4_ emissions during the growing season, with only weak CH_4_ absorption (–0.01 to –0.007 mg m^–2^ h^–1^) during the non-growing season. Notably, there was a significant difference in soil CH_4_ flux between the clay loam of the Sihong poplar plantation and the sandy loam of the Dongtai poplar plantation. From August to December 2019 and from July to August and November 2020, the soil CH_4_ flux in the Sihong poplar plantation was significantly higher than in the Dongtai poplar plantation. Moreover, the soil CH_4_ flux significantly increased with rising soil temperature and soil water content. Diurnally, the soil CH_4_ flux followed a unimodal variation pattern at different growing stages of poplars, with peaks occurring at noon and in the afternoon. However, the soil CH_4_ flux did not exhibit a consistent seasonal pattern across different years, likely due to substantial variations in precipitation and soil water content. Overall, our study emphasizes the need for a comprehensive understanding of the spatiotemporal variations in forest soil CH_4_ flux with different soil textures. This understanding is vital for developing reasonable forest management strategies and reducing uncertainties in the global CH_4_ budget.

## Introduction

Methane (CH_4_) ranks as the second most significant anthropogenic greenhouse gas, following carbon dioxide (CO_2_), and its presence in the atmosphere has shown a swift and notable rise during recent decades^1^. After atmospheric consumption by OH radicals, soil methanotrophs represent the second most substantial CH_4_ sink, ranging between 20 and 45 teragrams (Tg) of CH_4_ per year^2^. Forested upland soil exhibits CH_4_ uptake of 13.88 (9.18–17.7) Tg per year, constituting roughly 43% of the aggregate soil CH_4_ consumption^2–4^. However, the CH_4_ consumption in forest soil is not always stable. Changes in temperature and hydrology may cause forest soil to transition from a CH_4_ sink to a CH_4_ source^5,6^. Poplar (*Populus* spp.) is globally widespread and known for its rapid growth and effortless hybridization within and between species^7^. Globally, poplar trees cover an extensive 31.4 million hectares^8^, with a notable presence covering 8.25 million hectares in China^9^. Remarkably, poplar plantations emerge as a distinctive forest type susceptible to temperature and hydrology variations^10^. Therefore, tracking the soil CH_4_ flux in these extensively dispersed and fast-growing plantation forests is crucial for precise estimation of the CH_4_ budget within the forest ecosystem.

Soil CH_4_ exchange dynamics are shaped by two contrasting biological processes: anaerobic methanogenesis and aerobic methanotroph consumption^11,12^. Nonetheless, substantial ambiguity exists regarding the scale and temporal variations in CH_4_ emissions from forests. The net CH_4_ flux results from a complex interaction of factors affecting diffusion and soil oxygen levels^13^, including soil texture, structure, temperature, and moisture^14,15^. Several investigations have proposed that variations in soil temperature, soil air-filled porosity, and soil physical properties (e.g., texture) have the potential to influence the extent of CH_4_ uptake. However, these factors typically cannot induce a shift in the direction of CH_4_ flux from a sink to a source^16,17^. Soil gas diffusion depends on soil texture and structure, which subsequently impacts the activity and scale of soil microbial communities engaged in gas diffusion or CH_4_ transformation^18,19^. Sandy soils are generally more efficient at oxidizing CH_4_ than silty soils due to their better ability to diffuse gases^20,21^. In contrast, the gas diffusion capacity of clay loam is poorer than that of sandy loam, making it easier to form an anaerobic environment conducive to CH_4_ production^22^. Nevertheless, only a small amount of research has been conducted on the impact of soil texture on CH_4_ flux. Therefore, more on-site measurements are crucial for narrowing our knowledge gap.

The contribution of forest CH_4_ flux to the greenhouse gas budget remains surrounded by substantial uncertainty^23^. Micro-meteorological assessments of CH_4_ fluxes within forested ecosystems have revealed that certain upland canopy environments exhibit a net CH_4_ uptake^24^, whereas others function as sources of CH_4_ emissions over annual cycles or specific time intervals^25,26^. This variability could arise from intermittent CH_4_ emissions from soil in humid regions, potentially enabling canopy-level CH_4_ emissions^23^. The primary source of these uncertainties lies in the pronounced spatiotemporal heterogeneity of CH_4_ flux^27^. The temporal dynamics of soil-atmosphere greenhouse gas exchange exhibit variations following daily and seasonal patterns^28,29^. These patterns are mainly modulated by shifts in soil moisture content and temperature^30^. Fluctuations in precipitation and air temperature across space and time modify soil moisture and temperature dynamics, consequently influencing soil CH_4_ flux^31^.

The extrapolation of CH_4_ flux data from traditional static chambers to the stand level suggests that upscaling CH_4_ flux using the static chamber approach does not align with ecosystem-scale CH_4_ flux observed through eddy covariance (EC) methods^23,32^. Monitoring CH_4_ fluxes at the ecosystem scale using EC techniques has comprehensive advantages, including long-term time series, large spatial scale, and consideration of the relationship between CH_4_ fluxes and changes in environmental factors^33^. However, the EC technique is highly dependent on weather conditions and may experience gas leakage due to topography, along with limited precision in tracking minimal CH_4_ flux levels^34,35^. Static chamber measurements are an effective method for monitoring low throughput and can capture CH_4_ emissions at “hot spots” and “hot moments”. Although CH_4_ fluxes have been assessed across global forest ecosystems, only a few studies extensively track the diurnal, seasonal, and interannual changes in CH_4_ emissions at the soil surface level. This constraint obstructs our comprehension of the temporal changes in soil CH_4_ fluxes and the spatiotemporal patterns of CH_4_ fluxes arising from subtle shifts in environmental factors such as temperature, precipitation, and soil moisture.

Our primary objectives were to explore the temporal and spatial patterns governing soil CH_4_ flux at the ground scale in poplar plantations, as well as to identify the relationship between key environmental drivers and soil CH_4_ flux. We hypothesized that: (1) the soil CH_4_ flux in poplar plantations exhibits distinct diurnal and seasonal patterns, and (2) CH_4_ flux in soils with sandy loam texture would be lower in magnitude compared to soils with clay loam texture during the same periods.

## Results

### Patterns of temperature, precipitation, and soil water content in two poplar forests

**Figure 1:**
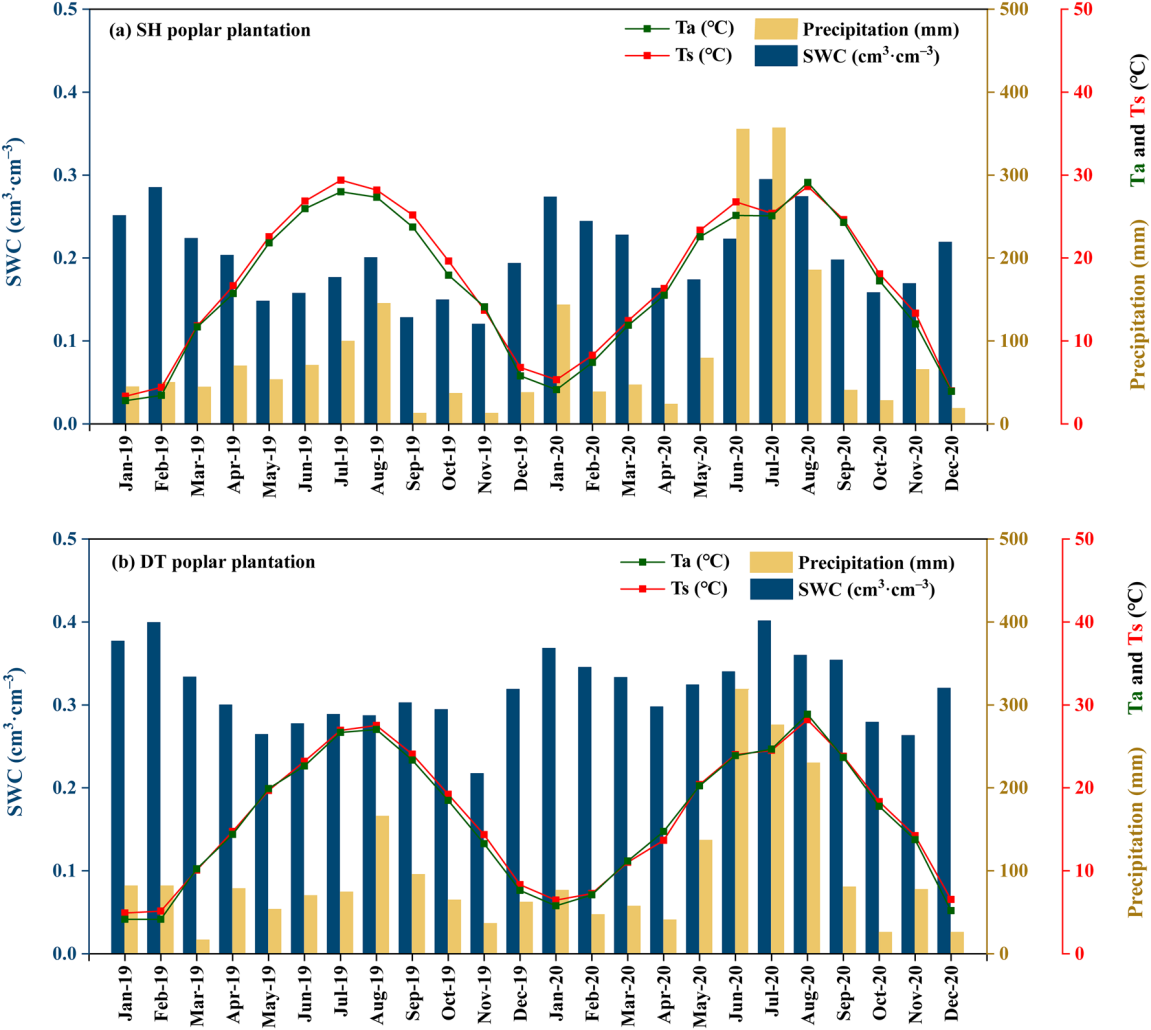
Monthly averages of air temperature, cumulative precipitation, soil temperature, and soil water content in Sihong (**a**) and Dongtai (**b**) poplar plantations. SH, Sihong; DT, Dongtai.Ta, Ts, and SWC represent air and soil temperature and soil water content.

Throughout 2019–2020, trends in Ta, precipitation, Ts, and SWC exhibited consistent patterns in both the Sihong and Dongtai poplar plantations (Fig. 1a,b). In 2019, the highest temperatures in the Sihong and Dongtai poplar plantations were recorded in July (28.0 ℃ for Ta and 29.4 ℃ for Ts) and August (27.0 ℃ for Ta and 27.5 ℃ for Ts), respectively. In 2020, the peak temperatures in both plantations occurred in August, with Sihong reaching 29.1 ℃ for Ta and 28.6 ℃ for Ts, and Dongtai reaching 28.9 ℃ for Ta and 28.2 ℃ for Ts. Precipitation in both plantations exhibited a unimodal pattern. In 2019, the peak monthly cumulative precipitation occurred in August, with 145 mm in Sihong and 166 mm in Dongtai. In 2020, the peak monthly cumulative precipitation in both plantations occurred in July for Sihong (357 mm) and June for Dongtai (319 mm).

Throughout 2019–2020, the SWC in the Dongtai poplar plantation consistently exceeded that in the Sihong poplar plantation, likely due to slightly higher precipitation levels in Dongtai compared to Sihong. Generally, the SWC in both poplar plantations exhibited a seasonal trend, with lower levels during summer and higher levels during winter. Notably, during the summer of 2020, increased rainfall resulted in the SWC reaching its peak in July for both the Sihong and Dongtai poplar plantations, recording values of 0.29 and 0.40 cm^3^ cm^–3^, respectively.

### Diurnal variations of soil CH_4_ flux in two poplar forests

**Figure 2:**
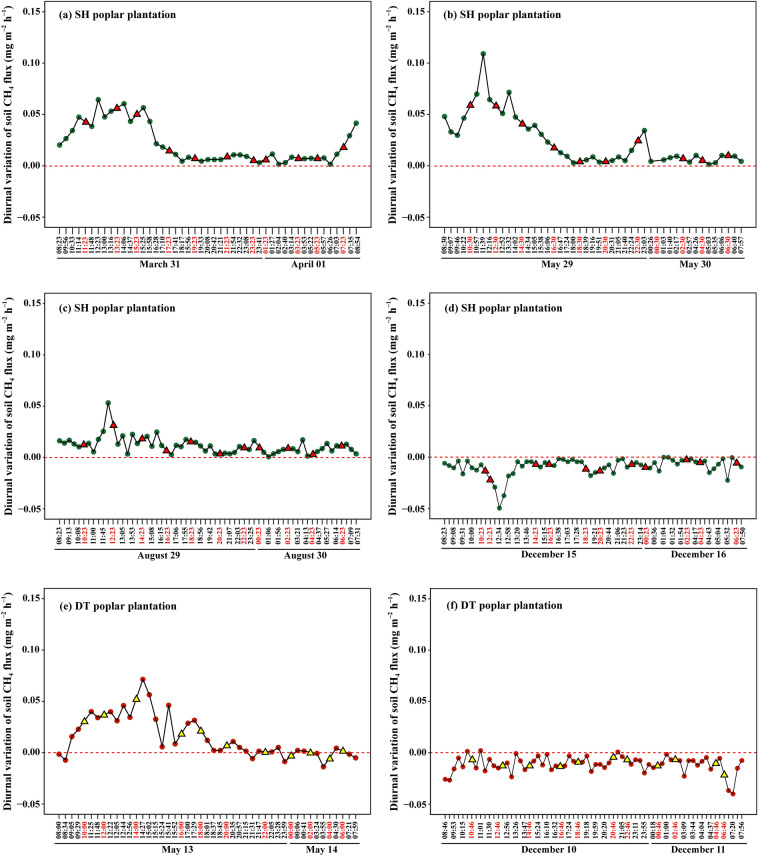
Diurnal variation of soil CH_4_ fluxes in the Sihong (**a**–**d**) and Dongtai (**e**–**f**) poplar plantations at different growing periods in 2019. Positive and negative values of CH_4_ flux represent the CH_4_ emission and uptake, respectively. The triangles represent the flux values marked at 2-hour intervals. SH, Sihong; DT, Dongtai.

In the Sihong poplar plantation, during the early growing season (March 31 to April 1) and the rapid growing season (May 29 to 30), the soil exhibited CH_4_ emissions throughout the day (ranging from 0.001 to 0.11 mg m^–2^ h^–1^), with peak emission rates observed at around 12:30 (0.06 and 0.11 mg m^–2^ h^–1^), followed by a gradual decline. From 18:00 until the next day at 08:00, the emission rate remained stable (Fig. 2a,b). During the late peak growing season (August 29 to 30), the soil emitted CH_4_ throughout the day (0.001 to 0.05 mg m^–2^ h^–1^), peaking at around 12:00, and then exhibited a fluctuating and decreasing trend (Fig. 2c). During the non-growing season (December 15 to 16), the soil did not emit CH_4_ but instead showed CH_4_ absorption throughout the day (–0.05 to –0.0001 mg m^–2^ h^–1^), with fluctuations and a peak absorption rate at 12:30 (Fig. 2d).

Due to logistical challenges related to transportation and weather conditions, we were only able to obtain effective diurnal variation data for the Dongtai poplar plantation during the rapid growing season (May 13 to 14) and the non-growing season (December 10 to 11) (Fig. 2e,f). During the rapid growing season, the soil CH_4_ emission rate increased from the early morning and reached a peak of 0.07 mg m^–2^ h^–1^ at 14:30. It then gradually decreased until 18:00 and remained relatively stable until the next day at 08:00. Notably, the soil exhibited CH_4_ emissions during the daytime (0.0007 to 0.07 mg m^–2^ h^–1^), while showing fluctuations between emission and absorption during the nighttime (–0.02 to 0.02 mg m^–2^ h^–1^). During the non-growing season, the soil absorbed CH_4_ throughout the day (–0.04 to –0.0007 mg m^–2^ h^–1^), with fluctuations trend, reaching a peak absorption in the morning before sunrise.

### Seasonal variations of soil CH_4_ flux in two poplar forests with different soil textures

**Figure 3:**
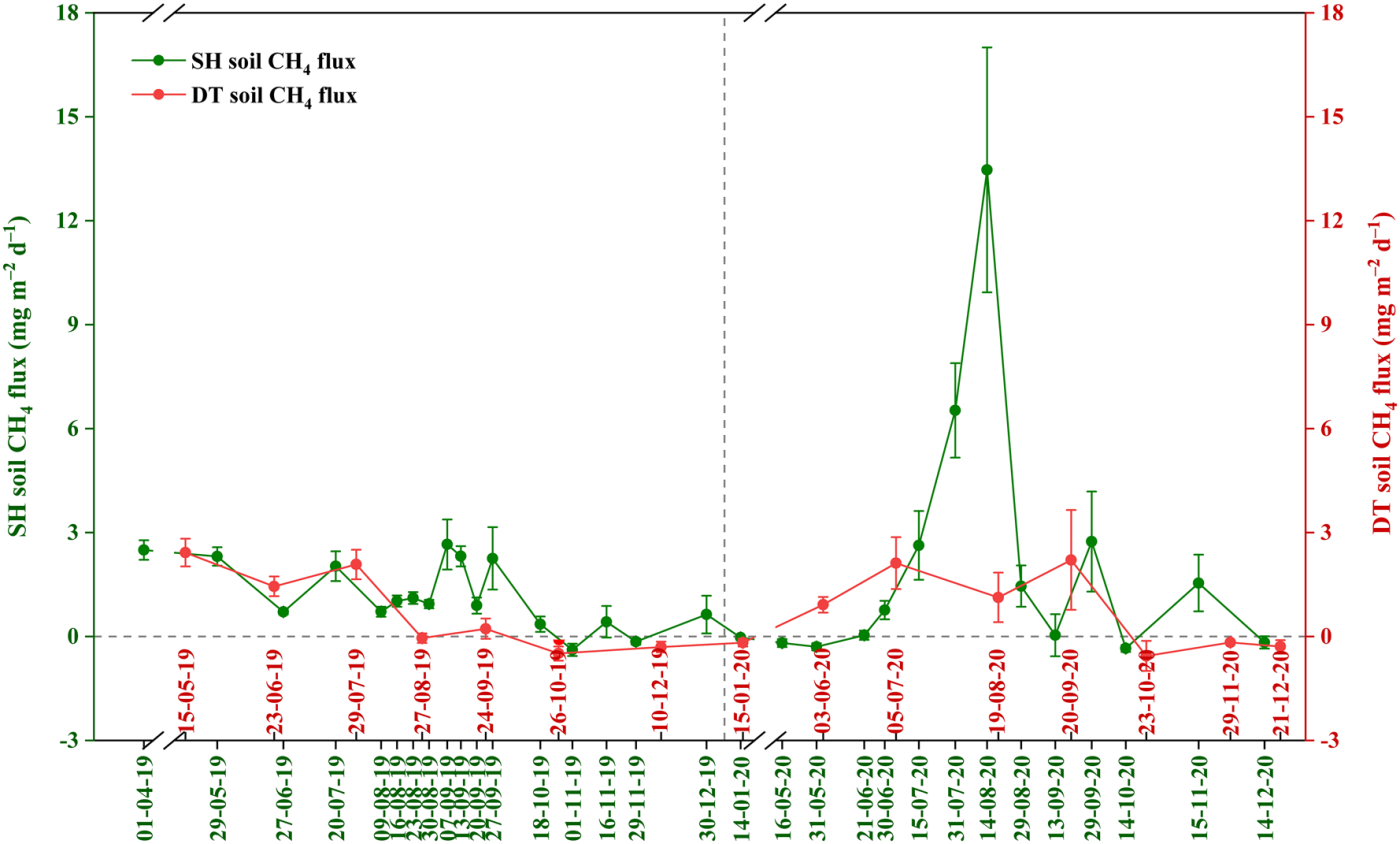
Daily average soil CH_4_ flux at different sampling days during the study period April 2019–December 2020 in the Sihong and Dongtai poplar plantations. Positive and negative data of CH_4_ flux stand for the CH_4_ emission and uptake, respectively. Error bars represent the standard error. Data from February 1 to April 30, 2020, was not gathered due to the COVID-19 pandemic. SH, Sihong; DT, Dongtai.

From April 2019 to December 2020, we conducted 31 CH_4_ sampling events at the Sihong poplar plantation, with a daily average soil CH_4_ flux of 1.56 ± 0.50 mg m^–2^ d^–1^ (Fig. 3). In the Dongtai poplar plantation, we conducted 15 sampling events, resulting in a daily average soil CH_4_ flux of 0.70 ± 0.39 mg m^–2^ d^–1^. Both plantations exhibited CH_4_ emissions during the growing season and a weak CH_4_ uptake during the non-growing season. From April to December 2019, the soil CH_4_ flux in both plantations fluctuated slightly and showed a decreasing trend over time. However, in 2020, the soil CH_4_ flux in both plantations displayed a unimodal seasonal variation pattern, with a peak occurring between July and September.

**Figure 4:**
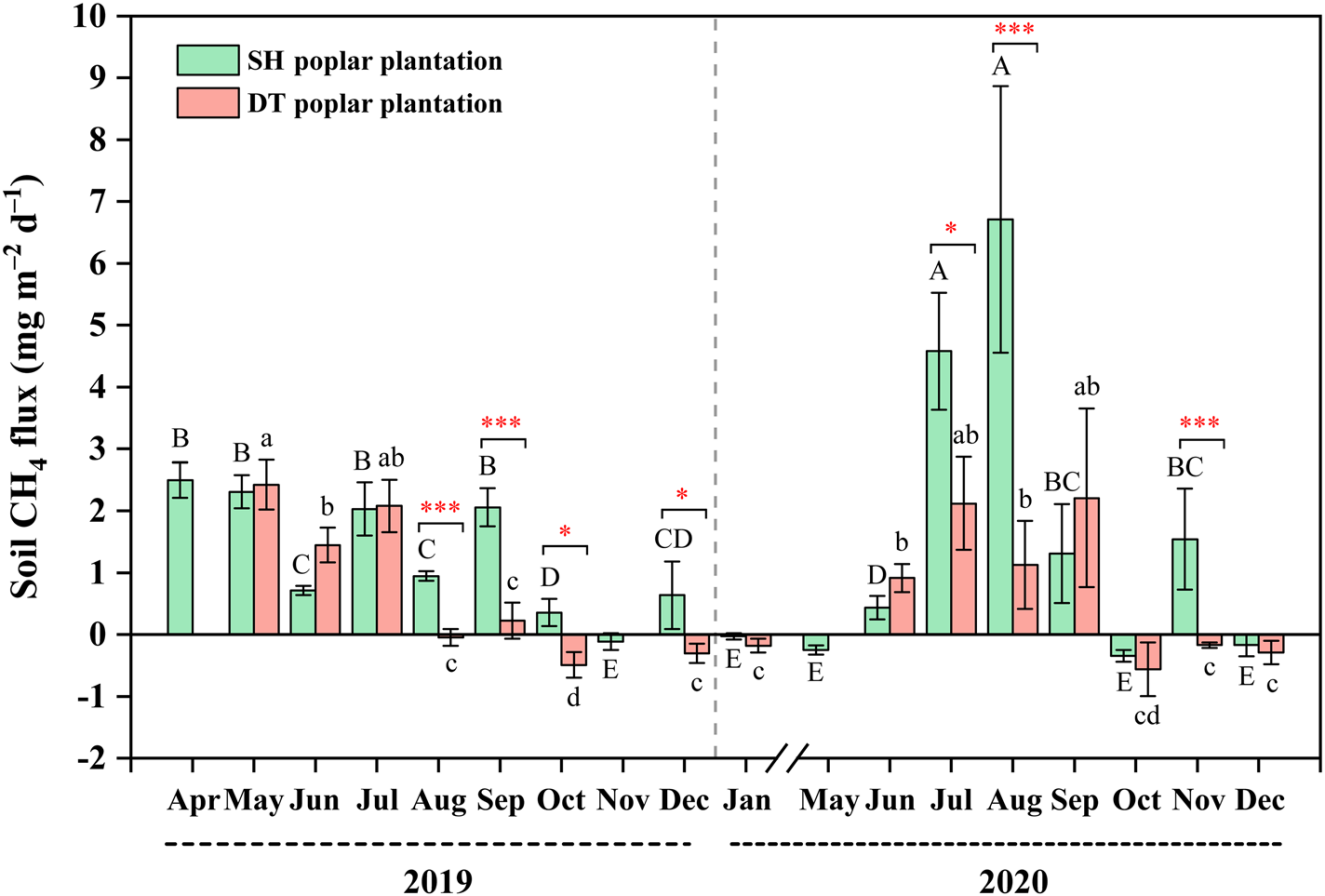
Spatiotemporal differences of monthly average soil CH_4_ flux in the Sihong and Dongtai poplar plantations. Positive and negative data of CH_4_ flux stand for the CH_4_ emission and uptake, respectively. The capital and lower-case letters on the error bars represent respectively the significant difference (at *P* ≤ 0.05) of soil CH_4_ fluxes in the Sihong and Dongtai poplar plantations at different months. Asterisks indicate the significant difference in soil CH_4_ fluxes between the Sihong and Dongtai poplar plantations in the same month (* 0.01 < *P* ≤ 0.05, ** 0.001 < *P* ≤ 0.01, * *P* ≤ 0.001). Error bars represent the standard error. Data from February 1 to April 30, 2020, was not gathered due to the COVID-19 pandemic. SH, Sihong; DT, Dongtai.

A *t*-test revealed that the soil CH_4_ fluxes in the Sihong poplar plantation significantly exceeded those of the Dongtai poplar plantation from August to October and December 2019, as well as July to August and November 2020 (Fig. 4). Repeated measure analysis indicated that, in the Sihong poplar plantation, the soil CH_4_ fluxes in April, May, July, and September 2019 were significantly higher than those in June and August. Furthermore, soil CH_4_ fluxes in June and August were significantly elevated compared to October and November, with July and August 2020 also displaying significantly higher CH_4_ fluxes compared to January, May, June, and September to December. In the Dongtai poplar plantation, the soil CH_4_ fluxes from May to July 2019 were significantly higher than those from August to December. Moreover, soil CH_4_ fluxes between June and September 2020 exhibited a significant increase compared to January and the period from October to December.

### Relationships between soil CH_4_ fluxes and environmental factors in two poplar forests

**Figure 5:**
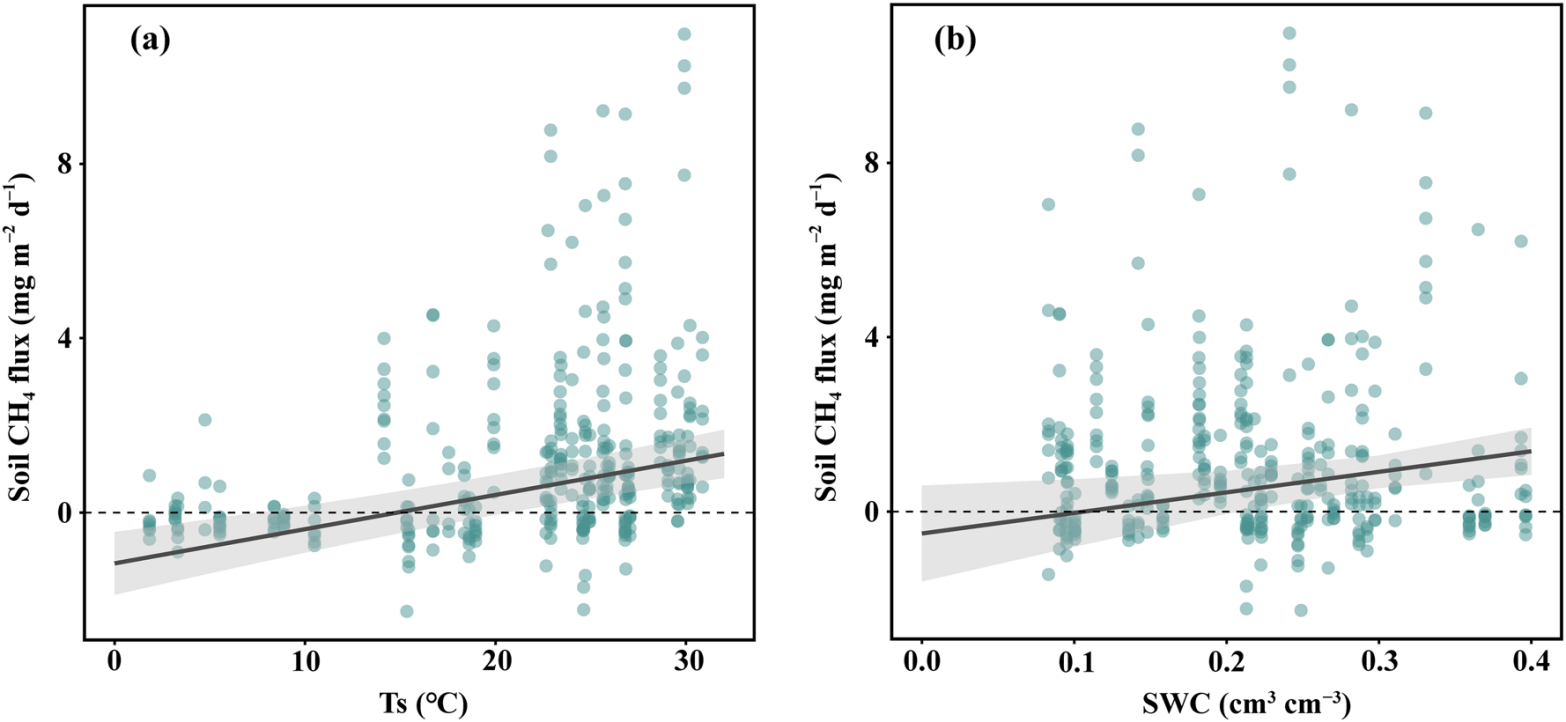
The effect of soil temperature (**a**) and soil water content (**b**) on the soil CH_4_ flux. The light gray shading indicates the 95% confidence interval. Ts, soil temperature; SWC, soil water content.

To identify the dominant driving factors influencing changes in CH_4_ fluxes between the soil and the atmosphere, we used a GLMM to test the effects of environmental factors on CH_4_ fluxes. The result indicated that soil CH_4_ fluxes significantly increased with rising Ts (*P* = 0.010) and SWC (*P* < 0.001) (Table 1; Fig. 5). However, precipitation did not have a significant impact on soil CH_4_ fluxes (*P* = 0.634).

**Table 1:** Statistical summary of generalized linear mixed model analysis of the effect of climatic and soil factors as fixed effects and soil static chambers as random effect on soil CH_4_ fluxes.

	Soil CH_4_ flux		
Predictors	Estimates	95%CI	*P*
SWC	4.721	1.156–8.287	**0.010**
Ts	0.079	0.051–0.106	** < 0.001**
Precipitation	0.027	− 0.084–0.137	0.634
Site [DT]	− 2.202	− 3.460–− 0.944	**0.001**
Site [SH]	− 1.254	− 2.215–− 0.292	**0.01**
*Random Effects*			
σ^2^	3.60		
τ_00 Chamber_	0.00		
N _Chanber_	18		
Observations	339		
Marginal *R*^2^	0.140		

Numeric variables such as daily average of Ts, SWC, and precipitation were standardized, that is, subtract the mean and then divide by the standard deviation. The bold *P*-values indicate the significant impacts of fixed factors on soil CH_4_ fluxes (*P* < 0.05). Ts, soil temperature; SWC, soil water content. SH, Sihong; DT, Dongtai.

## Discussion

In this investigation, diurnal patterns displayed a single emission peak during the growing season, specifically in its early stages (March and May), occurring between 12:30 and 14:30. During the non-growing season (December), there was a solitary uptake peak detected at 12:30. (Fig. 2). These findings support our first hypothesis regarding diurnal variation trends. However, our results differ from previous observations in upland forests, which generally indicated an uptake peak around noon during the summer^36,37^ (Table 2). Xiao et al. reported a bimodal diurnal CH_4_ uptake pattern in a deciduous forest that persisted across all seasons, featuring peaks at 14:00 and 18:00, respectively ^38^. Although their diurnal patterns of uptake or emission varied, a commonality among these studies is that diurnal variations are primarily driven by soil temperature. In two other studies, no clear regularity was found during the measurement of diurnal variation in March and May^39,40^. Furthermore, research revealing the diurnal cycles of ecosystems using the EC method also showed various patterns. For example, Querino et al. reported a single emission peak throughout the year in a tropical forest, peaking in the morning after sunrise^41^. In a subtropical forest, Wang et al. observed a singular uptake peak in summer, a lone emission peak in winter, and an absence of a consistent pattern in both spring and fall^42^. Overall, the diurnal cycles exhibited diversity in different ecosystems, driven mainly by temperature.

**Table 2:** Comparison of the diurnal and seasonal patterns of methane flux for various different forest types.

Forest type	Location	Diurnal variation	Season variation	Correlated factors	Method	Scales of flux	Source
Temperate forest	Central Japan (34°56′-35°22′N, 135°54′-136°16′E)	Uptake (small and not periodic)	Higher from late summer to autumn	No correlation with soil temperature	Static chamber	Soil	^39^
Subtropical forest	Gongga Mountain (29°00′-30°20′N, 101°30′-102°15′E)	Uptake (fluctuate drastically with unclear regularities)	Fluctuate drastically with unclear regularities (peaked at May–July, and/or September)	No correlation with air and soil temperature	Static chamber	Soil	^40^
Temperate forest	Changbai Mountain (42°24′N, 128°28′E)	Uptake (Bimodal)	The highest uptake occurred in summer and autumn (peaked at June and September)	Soil temperature	Static chamber	Soil	^38^
Temperate forest	Greater Khingan Mountains (50°49' ~ 50°51'N, 121°30' ~ 121°31'E)	Uptake (single peak)	Single peak at August	Soil temperature and moisture	Static chamber	Soil	^36^
Temperate forest	Central Ontario (45°17′11″N, 78°32′19″W)	Uptake	Uptake increasing from June to September and remaining high in October	Temperature for diurnal cycle; Soil water content control the seasonal changes	Static chamber	Soil	^43^
Tropical montane rainforest	Jianfengling National Nature Reserve (18°23′ ~ 18°50′N, 108°36′ ~ 109°05′E)	Uptake (single peak)	/	Soil temperature	Static chamber	Soil	^37^
Tropical forest	Manaus, Brazil (2°36′32.67″S, 60°12′33.48″W)	Emission (single peak)	/	/	Eddy covariance (EC)	Ecosystem	^41^
Subtropical forest	Tianmu Mountain Nature Reserve (30°20′34.951″N, 119°26′08.671″E)	Uptake and emission (both single peak)	Uptake in summer and emission in winter	Soil temperature and moisture for seasonal changes	EC	Ecosystem	^42^
Temperate forest	Fluxnet-Canada Research Network (54.95384°N, 112.46698°W)	Emission (single peak)	Emission peak in late July (single peak)	Solar radiation, latent heat flux, air temperature and ecosystem conductance to water vapor	EC	Ecosystem	^58^

Despite the soil serving as a CH_4_ sink during some non-growing season periods, the net emissions during periods of high temperature and humidity in the growing season were substantial enough to compensate for these limited CH_4_ sinks. This contrasts with some previous studies that have reported forest soils acting as a CH_4_ sink. In some temperate forests, previous studies have revealed that the soil functions as a CH_4_ sink, especially showing higher uptake in summer and autumn^36,38,39^. In three subtropical forest ecosystems in southern China, the soil was identified as a CH_4_ sink, demonstrating an annual average CH_4_ uptake of 3.4 ± 0.9 kg CH_4_-C ha^–1^ yr^–1^^29^. In a recent global synthesis, Feng et al. found that nearly 117 M ha of forest soils emit CH_4_ gas with an average magnitude of 0.84 ± 0.83 kg CH_4_-C ha^–1^ yr^–1^^3^. Many previous studies found that higher fluxes generally occurred in summer and autumn^36,38,39^, suggesting temperature plays a critical role in regulating soil CH_4_ dynamics. The results of the GLMM in this study further support this view (Table 1). The soil CH_4_ flux significantly increased with rising soil temperature (Fig. 5a).

The soil CH_4_ fluxes from the Sihong and Dongtai poplar forests in 2019 did not exhibit a specific seasonal pattern, aligning with earlier research results^29,40^. However, in 2020, the soil CH_4_ flux within these poplar plantations displayed a clear unimodal seasonal pattern, with peak fluxes occurring in July–August (Sihong) and July–September (Dongtai), markedly surpassing those observed in other seasons. The seasonal pattern of a single peak observed in our findings is consistent with other studies^36,38,43^. The observed variation in seasonal fluxes between years could potentially be attributed to interannual fluctuations in the summer hydrologic cycle, including factors such as rainfall and soil water content (Fig. 1). The GLMM showed that the soil CH_4_ flux significantly increased with rising soil water content (Fig. 5b). The net CH_4_ fluxes could be frequently influenced by precipitation and soil moisture conditions^28^. Particularly under the conditions of the Asian monsoon climate, CH_4_ emissions could increase with the frequency and amount of summer rainfall^44^.

According to previous studies, soil texture has been considered a strong predictor of CH_4_ fluxes^2,45^. In this study, we found that CH_4_ emissions from the Sihong poplar plantation, which had a clay loam soil texture, were significantly greater during the periods of August to December 2019 and July to August and November 2020 when compared to the emissions from the Dongtai poplar plantation with a sandy loam soil texture during the same time intervals (Fig. 4). Prior research has highlighted the importance of soil texture in governing greenhouse gas emissions through its influence on the quantity of micro- and macropores within the soil, which is essential for aeration and subsequently affects gas diffusion^28^. Increased clay content could hinder the diffusion of atmospheric CH_4_ into the soil, restricting aerobic CH_4_ oxidation and enhancing anaerobic microsite CH_4_ production in clayey soils, particularly during the rainy season^46,47^. Additionally, Martins et al. highlighted that soil moisture and sand content exerted the most pronounced direct influence on CH_4_ fluxes, while rainfall indirectly affected fluxes by directly impacting soil moisture and gas diffusivity^48^. Therefore, in our study, soil texture could be the main factor directly influencing soil CH_4_ fluxes, considering the climate conditions at the two sites have no significant difference.

Considering the significant impact of soil texture on CH_4_ flux, altering the soil composition within poplar plantations presents a viable strategy for emission reduction. For example, introducing organic matter or adjusting soil composition to enhance drainage and aeration can effectively reduce the creation of anaerobic conditions that foster methanogenesis. Fine-textured soils, such as clay loam, may be amended with coarser materials, such as harvesting residuals, to improve gas diffusion and promote aerobic conditions. Furthermore, modifying soil properties through microbial manipulation has the potential to influence CH_4_ flux. Incorporating organic matter, like compost or biochar, can augment microbial activity and foster aerobic conditions. An emerging area of study involves utilizing specific methane-oxidizing microbes to enhance CH_4_ uptake. Although this study has shed light on CH_4_ spatiotemporal dynamics within poplar plantations, it has certain limitations, such as the focus on only two sites and the use of spark measurements with the chamber-GC approach. Addressing these limitations through extended, long-term monitoring, broader geographical coverage, and more comprehensive factor analysis is necessary to gain a deeper understanding of soil CH_4_ flux in plain forests.

## Conclusion

Our study contributes to the understanding of spatiotemporal dynamics of soil CH_4_ flux in two poplar plantations on plains. At both plantation sites, the soil exhibited net CH_4_ emissions throughout the growing season while demonstrating weak CH_4_ uptake during the non-growing season. The high CH_4_ emissions during periods of high temperature and humidity offset the slight CH_4_ sinks. Sandy loam soil displays higher CH_4_ absorption rates in winter, whereas clay loam soil exhibits greater CH_4_ emissions. On a daily scale, there are significant absorption peaks in winter and significant emission peaks in other seasons in both forests, primarily driven by temperature. On a seasonal scale, the variation of soil CH_4_ flux primarily depended on differences in temperature and soil water content. Overall, our study provides valuable insights into multi-time-scale CH_4_ measurements on the soil surface and highlights the importance of considering temporal dynamics to reduce the deviations in the CH_4_ budget. These findings contribute to improving the accuracy of ecosystem CH_4_ budget assessments, aiding in the management and mitigation of greenhouse gas emissions in plain forests. Future studies can build upon these findings to develop more accurate models and strategies for mitigating CH_4_ emissions in such forests.

## Materials and methods

### Site description

The study was conducted at two distinct sites: the Sihong poplar plantation (33°19′20′′ N, 118°18′30′′ E) located on the western shore of Hongze Lake, covering an area of 800 ha in the Huang-Huai Plain, and the Dongtai poplar plantation, covering an area of 3,000 ha (32°51′26′′ N, 120°51′01′′ E), a significant coastal protection forest in the middle and lower reaches of the Yangtze River Plain, China^49^. The four seasons of spring, summer, autumn, and winter in these two research areas refer to March to May, June to August, September to November, and December to February of the following year, respectively. The Sihong poplar plantation is bordered by water on three sides and falls within the bounds of a characteristic subtropical monsoon climate. The area has a mean annual sunlight duration of 2,327 hours, a mean annual temperature (MAT) of 14.1 ℃, and a mean annual precipitation (MAP) of 897 mm. The soil is classified as Hongze Lake silted soil, identified as Gleysols following the IUSS World Soil Classification^50^, with a clay loam texture. It comprises 410, 330, and 260 g kg^–1^ of silt, sand, and clay, respectively. The Sihong poplar plantation was established in 2007 using 1-year-old seedlings of the ‘Nanlin-95’ clone, a hybrid derived from the combination of clone I-45 (*P*. *euramericana* [Dode] Guineir. cv. ‘I-45/51’) and clone I-69 (*Populus deltoides* Bartr. cv. ‘Lux’). The understory vegetation mainly comprises herbs, including *Solidago canadensis*, *Erigeron pseudotenuicaulis*, *Perilla frutescens*, *Geranium wilfordii*, and *Duchesnea indica*.

The Dongtai poplar plantation is situated along the coastline of the Yellow Sea and falls within a marine monsoon climate zone. It has an average annual sunshine duration of 2,209 hours, a MAT of 14.6 ℃, and a MAP of 1,050 mm. The soil in this region is classified as desalinated meadow soil, categorized as Fluvisols according to the IUSS World Soil Classification^50^, and is characterized by a sandy loam texture. The soil consists of 160, 720, and 120 g kg^–1^ of silt, sand, and clay, respectively. The Dongtai poplar plantation, initiated in 2006, consists of a single-cultivar plantation utilizing *P. canadensis* cv. ‘I-72/58’. The understory vegetation consists of a few species of shrubs dominated by *Morus alba* and *Broussonetia papyrifera*, as well as herbs including *Solidago canadensis*, *Cayratia japonica*, *Microstegium vimineum*, *Carpesium abrotanoides*, *Achyranthes bidentata*, and *Rostellularia procumbens*. We did not intervene or manage litter in the two forests during the entire experimental period.

**Figure 6:**
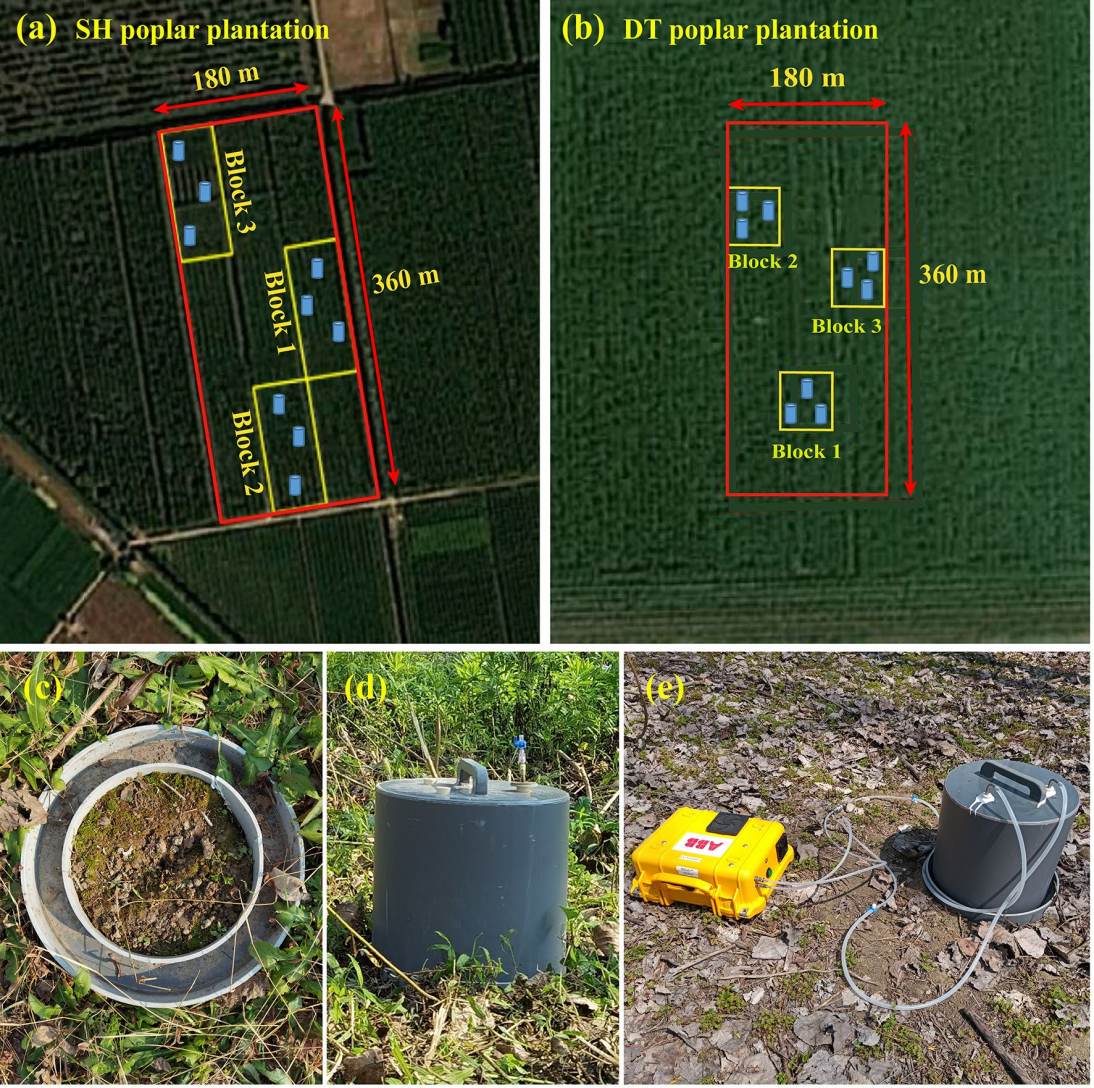
Layout of experimental design for the Sihong (**a**) and Dongtai poplar plantations (**b**), static chamber base installation (**c**), static chamber seal (**d**), and diurnal variation sampling of soil CH_4_ flux (**e**). SH, Sihong; DT, Dongtai.

Three sample plots were established as replicates at both sites, respectively, with each plot measuring 60 m × 120 m in Sihong and 60 m × 60 m in Dongtai (Fig. 6a,b). In March 2019, before gas sampling, five points were chosen per block using an S-type pattern to collect soil samples at a 0–20 cm depth for measuring soil physical and chemical characteristics. Soil samples from the five points within each block were uniformly blended to create an independent sample. The fresh soil samples were brought to the lab in sterile fresh-keeping bags and reserved at 4 ℃. After removing plant roots and small stones, some samples were left to air-dry naturally and subsequently sifted using a 2 mm mesh for further detection. Soil bulk density (BD) was assessed using the core method, while soil water content (SWC) was measured through the oven drying method (24-hour drying process at 105 ℃). Soil pH was determined by a pH meter (AB15 + Basic; Accumet, San Diego, CA, USA) with a 1:2.5 soil-to-water ratio, while soil organic carbon (SOC) was measured by potassium dichromate oxidation-ferrous sulfate titration. Total nitrogen content (TN) was measured using an automatic continuous flow analyzer (AA3; Bran Luebbe, Norderstedt, Germany) after digesting 1 g of dry soil with a catalyst (2 mL) and H_2_SO_4_ (5 mL). Ammonium nitrogen (NH_4_^+^-N) and nitrate nitrogen (NO_3_^−^-N) were determined through ultraviolet-visible (UV–vis) spectrophotometry (UV-2550; Shimadzu, Tokyo, Japan) following the extraction of 5 g of soil in 50 mL of 2 M KCl. Fifteen trees in each block were selected to measure tree height and diameter at breast height (DBH). Tree height was measured using vertex laser rangefinders (Haglöf, Langsele, Sweden), while DBH was measured by a caliper. The stand characteristics and basic soil physicochemical properties of the Sihong and Dongtai poplar plantations are detailed in Table 3.

**Table 3:** Stand characteristics and soil properties (0–20 cm) in the two investigated poplar plantations (mean ± SD, n = 3).

Indexes	Variables	Sihong poplar plantation	Dongtai poplar plantation
Stand characteristics	Stand age (yr^–1^)	15	16
	Mean tree height (m)	23.6 ± 2.2	20.1 ± 0.3
	Mean DBH (cm)	24.1 ± 2.4	24.0 ± 0.5
	Stand density (trees ha^–1^)	300	250
	Canopy density (%)	63	55
Soil physicochemical properties	BD (g cm^–3^)	1.31 ± 0.07	1.18 ± 0.06
	SWC (g kg^–1^)	206 ± 133	188 ± 155
	Soil pH	6.7 ± 0.1	7.8 ± 0.3
	SOC (g kg^–1^)	12.13 ± 1.8	8.25 ± 1.88
	TN (g kg^–1^)	0.96 ± 0.21	1.15 ± 0.29
	NO_3_^−^-N (mg kg^–1^)	6.65 ± 1.23	1.21 ± 0.44
	NH_4_^+^-N (mg kg^–1^)	23.5 ± 2.6	18.35 ± 12.81

DBH, Diameter at breast height; BD, Bulk density; SWC, Soil water content; SOC, Soil organic carbon; TN, Total nitrogen; NO_3_^−^-N, Nitrate nitrogen; NH_4_^+^-N, Ammonium nitrogen. SD, standard deviation.

### Soil CH_4_ gas collection and measurement

In March 2019, three random sampling points were selected within each block to collect soil CH_4_ gas samples in both plantations, respectively. Three cylindrical static chambers constructed from polyvinyl chloride (PVC) were randomly installed in each block in the Sihong and Dongtai poplar plantations, resulting in a total of 9 static chambers installed in each plantation (Fig. 6a,b). The static chamber system includes a base (30 cm inner diameter, 35 cm outer diameter, and 15 cm height) and a chamber (30 cm in both diameter and height). The base was inserted vertically into the ground to a depth of 10 cm, leaving 5 cm above ground, thereby creating a closed ring with distinct inner and outer diameters (Fig. 6c). During CH_4_ gas sampling, water was added to the rings to establish an effective seal between the base and the chamber (Fig. 6d). A stand (15 cm height) equipped with a temperature recorder (DS1923; Wdsen Electronic Technology Co. Ltd, Shanghai, China) was installed in the center of the base during the gas sampling period for calculating CH_4_ fluxes. Gas sampling was carried out at the center of the chamber top through a rubber stopper with a hole, using a syringe (18-gauge needle, 15 cm long) connected to a T-joint.

Specifically, from April 2019 to December 2020, gas sampling was conducted respectively for 9 chambers at the Sihong and Dongtai poplar plantations between 8:00 and 12:00 am on days without rain or snow. During each sampling day, we gathered 30 mL gas samples at intervals of 0, 20, 40, and 60 minutes and subsequently transferred them into pre-evacuated gas bottles for storage. At the Sihong poplar plantation, gas sampling frequency ranged from 1 to 4 times per month, resulting in a total of 31 on-site sampling activities and the collection of 1,116 soil gas samples. Due to traffic and accessibility constraints at the Dongtai poplar plantation, gas sampling was conducted once per month, resulting in 15 on-site sampling activities and the collection of 540 soil gas samples. Data collection was suspended from February to April 2020 owing to the impact of the COVID-19 pandemic.

Gas samples were transported to the lab and subsequently examined for CH_4_ concentration using a gas chromatograph (GC; 7890B, Agilent Technologies, Inc., Palo Alto, CA, USA). The GC used in the study was equipped with a pair of Porapak Q columns (each measuring 1.83 meters in length, with a 2 mm inner diameter and 80/100 mesh) alongside a flame ionization detector (FID). The column oven and detector were operated at temperatures of 60 ℃ and 250 ℃, respectively. We employed ultra-high purity nitrogen (N_2_, 99.999%) as the carrier gas, flowing at a rate of 30 mL min^–1^. Moreover, hydrogen (H_2_) and high-purity air (99.999%) served as the fuel and auxiliary fuel gases for the FID, with flow rates set at 40- and 400-mL min^–1^, respectively. The instrument was calibrated with a standard gas (10.2 ppm) both before and after each measurement. The formula for calculating soil CH_4_ flux^51^ (*F*, mg m^–2^ d^–1^) is as follows:

1 $$F=\rho \times \frac{V}{A}\times \frac{\Delta C}{\Delta t}\times \frac{273.15}{(273.15+T)}$$

Here, *ρ* represents the CH_4_ density under standard conditions (g L^–1^); *V* represents the volume of the static chamber (cm^3^); *A* denotes the surface area encompassed by the static chamber (cm^2^); and *T* stands for the temperature inside the static chamber during sampling moment (℃). Additionally, *∆C*/*∆t* represents the linear slope of CH_4_ concentration change within the chamber over time (ppm min^–1^), with an *R*^2^ ≥ 0.9 considered valid for further analysis. The limit of detection (LOD) of CH_4_ flux for the GC system was 0.49 ppm, calculated using methodologies provided by Minamikawa et al.^52^.

Moreover, air temperature (Ta) was measured using a platinum resistance thermometer (PRT) installed on a flux (CO_2_ and H_2_O) tower. Precipitation was recorded with a tipping bucket rain gauge installed at the base of the flux tower. For missing meteorological data, we gap-filled using data from the National Meteorology Information Center (NMIC) of China. Soil temperature (Ts) and SWC were measured using thermocouples and time domain reflectometry (TDR, Trime-EZ, IMKO) probes, respectively, installed at a depth of 10 cm.

### Measurement of diurnal variation of soil CH_4_

In 2019, we employed a swiftly deployable chamber system utilizing laser technology to measure diurnal variations in soil CH_4_ flux, using the Los Gatos Research (LGR; ABB, Canada) instrument. The recording frequency was set to 2 Hz (Fig. 6e). Monitoring took place during different growth stages of the poplar, including the early growing season (March–April), the rapid growing season (May–June), the late peak growing season (August–September), and the non-growing season (November–December). The measurement events were conducted on days without rain or snow in different seasons. Considering the complexities of field sampling, including the daytime heat, nighttime cold, and threats from certain animals (e.g., snakes), we selected a static chamber at each site to measure the diurnal variation of soil CH_4_ flux. The sampling started at around 08:00 am and ended the following day at around 08:00 am, with the static chamber sampled for 15 min each time. The CH_4_ concentration change rate per unit of time was calculated using the concentration values during the stable 10-minute period after excluding the first 3 min and the last 2 min of CH_4_ concentration values, with an *R*^2^ ≥ 0.9 considered valid for further calculating fluxes. There should be at least a 15 min interval between each measurement activity to ensure sufficient battery power and prevent the instruments from overheating and shutting down. The minimum detection limit (MDL) of the ultraportable LGR CH_4_ analyzer is 0.002 ppm, with an accuracy of less than 1%^53^.

### Statistical analysis

A repeated measures analysis of variance was used to examine variations in soil CH_4_ flux across different periods. Subsequently, a *T*-test was performed to evaluate the difference in soil CH_4_ flux between the Sihong and Dongtai poplar plantations for the same month. When the number of repetitions differed between the two groups, specific data processing steps were taken to ensure the validity of the comparison. For the Sihong site, where CH_4_ flux measurements were conducted two or four times in a month, the average of these replicates was calculated for each month and considered as a single monthly CH_4_ flux value. For the Dongtai site, where CH_4_ flux measurements were conducted only once per month, this single measurement served as the monthly CH_4_ flux value. A generalized linear mixed model (GLMM) was used to assess the effect of climate and soil factors on soil CH_4_ flux, incorporating precipitation, Ts, SWC, and sampling site (SH and DT poplar forests) as fixed effects and sampling points (static chambers) as a random effect. All numerical variables were standardized (subtracting the mean and then dividing by the standard deviation) to enhance the likelihood of model convergence^54^. We used the R package "performance" for model diagnostics to verify the normality of residuals and the multicollinearity of variables^55^. Variables displaying correlation coefficients exceeding 0.7 were eliminated from the analysis^56^. Unless otherwise stated, the statistical analysis was conducted at a significance level of 0.05. All statistical analyses were conducted using R software version 4.3.0^57^.

## Data Availability

Data available on request from the corresponding author.
